# Physical and mental stress assessment during robotic-arm assisted total knee arthroplasty

**DOI:** 10.1007/s11701-025-03017-6

**Published:** 2025-12-05

**Authors:** David Putzer, Adriana Palacio Giraldo, Julian Lair, Michael Nogler, Michael C. Liebensteiner, Martin Thaler

**Affiliations:** 1 Experimental Orthopaedics, Department of Orthopaedics and Traumatology, Medical University of Innsbruck, Anichstrasse 35, Innsbruck, 6020 Austria; 2 Division of Orthopedics and Traumatology, University Hospital Krems, Mitterweg 10, Krems, 3500 Austria; 3 Karl Landsteiner University of Health Sciences, Dr. Karl-Dorrek-Straße 30, Krems, 3500 Austria; 4 Private Hospital Mellitaklinik, Bolzano, Italy; 5 Orthopädie für Hüfte, Knie & Fuß im Zentrum, Innsbruck, Austria; 6 Private Hospital Kettenbrücke, Innsbruck, Austria; 7 Hospital St. Vinzenz Zams, Zams, Austria; 8 Center of Orthopaedics, Trauma Surgery and Rehabilitation Medicine, University of Greifswald, 17489 Greifswald, Germany; 9 Arthroplasty Center Munich West, Helios Klinikum, 81241 Munich, Germany

**Keywords:** Total knee arthroplasty, Robotic surgery, Intraoperative stress, Workload, Surgical team dynamics, Heart rate monitoring, Ergonomics

## Abstract

Robotic assistance in total knee arthroplasty (TKA) improves surgical precision but may alter intraoperative stress and workload among staff. This study evaluated these effects in 60 robot-assisted procedures involving surgeons, scrub technicians, circulators, and technicians. Preoperative stress was assessed using the STAI-6, intraoperative stress via heart rate, and postoperative workload, satisfaction, confidence, and team interaction through questionnaires. Preoperatively, most staff reported no anxiety, though newly introduced members showed severe anxiety in up to 15% of cases. Intraoperatively, stress patterns varied by role: surgeons peaked during implantation, scrub technicians and circulators during non-robotic phases, and technicians during robotic-specific steps, especially ligament balancing. The robotic arm did not increase surgeon stress but redistributed workload, reducing physical demands for scrub staff and circulators while raising responsibility for technicians. Postoperatively, satisfaction and confidence were high across groups, though scrub technicians reported the greatest workload from added robotic tasks. Robotic systems guided by dedicated professional personnel have the potential to reduce the intraoperative stress level of the whole surgical team, although it adds multiple additional steps to the traditional workflow.

## Introduction

Total knee arthroplasty (TKA) is the most commonly performed joint replacement worldwide [1, 2], with sharply rising utilization in the US and UK [3–6].TKA aims to reduce pain, restore functionality, and improve quality of life [1–3]. It can be performed using manual techniques or computer-assisted approaches, including robotic systems [4]. Robotic assisted total knee arthroplasty has proven to be a reliable procedure with reduced postoperative pain and more accurate implant positioning [5] that complements broader institutional goals of consistency, quality, and long-term cost-effectiveness [6].

Since the introduction of robot-assisted joint arthroplasty, research has primarily focused on the technology itself and patient outcomes [7]. However, the psychological and physical effects of robot use on surgical staff, especially surgeons, are limited [8].

The operating room is a complex, high-risk, and often stressful environment [14, 15], with key intraoperative stressors for surgeons including complications (e.g., bleeding), time pressure, distractions, workload, and the demands of new techniques and technologies [14].A substantial proportion of surgeons show symptoms of burnout—characterized by emotional exhaustion, depersonalization, and reduced professional satisfaction [16–19]. In a 2009 U.S. study, ~ 30% reported depressive symptoms and up to 40% showed signs of burnout [16], which negatively affects psychological, emotional, and physical well-being [18, 19].

Burnout in medicine arises from multiple factors, including increasing administrative demands, reduced time for patient care, long working hours, poor work–life balance, and the inherent pressures of the profession [18]. Surgical work adds further strain, as surgeons spend about 30% of their day in a highly complex, hazardous, and physically and psychologically demanding OR environment [14, 15]. Surgical performance is shaped by individual, patient-related, and environmental factors [20, 21], with stress recognized as a key contributor that adversely affects performance and patient safety [21–24].

Measuring stress in this area is extremely challenging, as stress can only be measured based on its subjective and objective effects, and many other factors affect medical staff in the operating room [9]. The absence of a standardized method for measuring stress has resulted in studies employing a wide range of markers, including heart rate (HR) [10], skin impedance [11], eye blink rate, cortisol levels [12], and subjective assessment tools such as the STAI-6 [10].

The introduction of a new surgical technique or technology is associated with a learning process to reach full proficiency, which can be quantified through a learning curve by tracking their experience over a period until competence in a new procedure is reached, and proficiency is comparable to that of a standard procedure [13–16]. The physical and mental stress experienced during this learning phase is usually not assessed.

This prospective study used self-reported measures of surgery to determine whether using a semi-active robot during robot-assisted TKA surgery adds an additional stress factor for surgical staff (surgeon, scrubtech, circulator, and technician). A combination of objective measurement methods (heart rate measurement) and subjective evaluation instruments (pre- and post-operative questionnaires) was used in this study. A key aspect of the study was the detailed comparison of robotic and non-robotic workflows.

## Materials and methods

A total of 81 consecutive robotic-arm-assisted surgeries were performed by three senior, Endocert-certified arthroplasty surgeons (each performing > 100 arthroplasties per year) [17], in a single-center study over 3 years from November 2018 to March 2022. The surgeons used the Triathlon Knee System and the Mako Robotic Arm Interactive Orthopedic (RIO) System (both from Stryker, Kalamazoo, MI, USA). One of the surgeons had extensive prior experience with navigation systems. The experience level of the scrub techs was not recorded; the technician had experience with 16 surgeries. 11 surgeries were excluded as no stress assessment was performed (first two surgeries, two surgeries in 2020, and 6 surgeries during the COVID pandemic due to personal restrictions in the surgical room). 10 surgeries were excluded due to incomplete data and or missing synchronization information, resulting in 60 datasets.

Ethical review and approval were waived for this study as no patient data was involved in the analysis and only staff specific parameters were recorded.

Stress assessment was performed on 19 TKAs for surgeon 1 (32%), 15 TKAs for surgeon 2 (25%), and 26 TKAs for surgeon 3 (43%). The same technician, except for one case, performed all cases. Ten scrub techs were involved in the study, each of them performing a minimum of four cases (median 6, range 4–8). In three cases, the scrub tech was switched during the procedure, and measurements were only taken for the first scrub tech. Questionnaires were recorded for all team members immediately before entering the surgical room or during the preparation phase of the surgery.

Current strategies for measuring stress include heart rate measurement [10], skin impedance [11], eye blink rate, and cortisol levels [12], as well as subjective assessment using the six-item State-Trait Anxiety Inventory (STAI-6) score [10, 18]. In this study, heart rate was measured to determine physical and mental stress, and the STAI-6 score was used to assess subjective stress levels before and after surgery for the surgical team (surgeon, scrub tech, and technician) [19]). The 6 items were based on a 5-point Likert scale: 1) calm, 3) tense, 6) upset, 15) relaxed, 16) content, and 17) worried. These items were described as the highest and the lowest anxiety items [18, 19], with a total score ranging from 6 to 30. The overall score was rescaled to 20–80, where scores ≤ 35 indicate no anxiety, 36–41 indicate moderate anxiety, and ≥ 42 indicate severe anxiety [18].

Heart rate (HR) measurements were taken from staff members (surgeons, scrub techs, technicians, and circulators) to evaluate biophysical stress and/or workload. An optical sensor (Polar OH1, sampling rate of 1 Hz, Kempele, Finland) was attached to the upper arm, just above the elbow, of the surgical staff. The sensor works using photoplethysmography to optically measure the volumetric changes (size) in blood vessels under the skin with the help of a light-emitting diode and a light detector [20].

Heart Rate Reserve (*HRR*) was calculated by considering the difference between maximum heart rate (*HR*_*max*_) and resting heart rate (*HR*_*rest*_):.

$$\:HRR={HR}_{max}-{HR}_{rest}$$

Any measured heart rate (*HR*_*meas*_) was normalized (*HR*_norm_) using the following formula and expressed as a percentage of an individual’s capacity:

$$\:{HR}_{norm}=({HR}_{meas}-{HR}_{rest})/HRR*100$$

In 47 cases, all three team members were equipped with an HR sensor. In six cases, only the surgeon and the technician wore the HR sensor. In five cases, only the scrub tech and technician wore the HR sensor. In one case, the surgeon and scrub tech wore the HR sensor. Four measurements in which only one team member was equipped with the sensor were excluded from the study. Two cases were excluded due to lost synchronization information, and four cases were excluded due to insufficient data. Additionally, 11 circulators were equipped with an HR sensor in 29 cases, with a median case number of 2 (range 1–5). In 25 datasets were all four members of the team were equipped with an HR sensor.

Preoperative CT based surgical planning was performed as well as standard planning based on x-rays and confirmed prior to the timeout within the surgery by the surgeon. In 7 cases (12%) no ligament balancing was performed, and the cuts for implant positioning were performed according to preoperative planning.

The cut to suture time from skin incision (first incision) to wound closure (end of suturing) was recorded and reported for each surgery. The surgical workflow was divided into 9 steps:

Incision and arthrotomy” includes all steps performed by the surgeon to expose the patient’s knee joint. Besides the standard surgical incision into the joint, up to four stab incisions are needed to place the femur and tibia arrays. The step “Bone registration” includes the placement of the navigation arrays and checkpoints, the collection of patient landmarks (hip center and medial and lateral maleoli), the collection of the bone surface on the femur and tibia, and the final verification process. During “Ligament balancing” the surgeon removes any osteophytes, which limit the movement of the knee joint, and records the joint space (the closest distance between the femoral surface and deepest point on the tibia surface in the medial and lateral compartments of the knee joint) in full knee extension and 90° of knee flexion. During this procedure, the surgeon applies tension to the knee joint in extension and flexion. Then, the surgeon instructs the technician to reposition the implant to adjust the varus/valgus deformity and laxity of the knee joint. In 3 cases, ligament balancing was not performed due to the surgeons’ preferences (5%). “Robotic-assisted bone resection”: The technician places the robot next to the patient’s operative side. Then, the surgeon verifies all necessary checkpoints and carries out the five necessary femur cuts (distal, posterior chamfer, posterior, anterior, and anterior chamfer) and a planar cut to the tibia. In the step “Preparation for implantation” the surgeon places the trial implants on the femur and tibia and performs a complete range of motion check. The medial-lateral alignment of the femoral implant, as well as internal-external rotation and anterior-posterior alignment, can be measured manually or with the navigation system. If the surgeon chooses the Triathlon posterior-stabilized (PS) implant, the PS box cut (intercondylar notch resection) must be performed without robotic assistance using the box guide. Distal fixation peg holes are drilled, keel preparation is performed using a keel punch, and if necessary, the patella preparation for the patellar implant will be carried out. Finally, the surgeon removes all trial implants and residual cartilage, cleans the cut edges, and washes away all debris, while the scrub tech prepares the bone cement in the meantime and the implants for the final implantation. In this study, all components were cemented in a single stage. “Implantation of components” was defined as the sequence of steps, including fixation of the femoral and tibial implants onto the prepared bone surfaces using bone cement, insertion and subsequent removal of a trial inlay, assessment of the range of motion, placement of the definitive inlay, and final verification of joint mobility. “Waiting for cement hardening” was defined as the period during which only minimal surgical manipulations were permitted while the limb was maintained in full extension, and the polymerization process of the polymethylmetacrylate was taking place. “Removal of navigation hardware” referred to the extraction of checkpoints and array pins that had been inserted to facilitate the robotic-arm-assisted procedure. “Suturing” was defined as the interval from the placement of the first stitch to the completion of wound closure.

The postoperative questionnaire comprised five items for each team member, each rated on a 5-point Likert scale. Questionnaires were recorded immediately after the surgery, before each team member left the surgical room. The items assessed satisfaction (“I am very satisfied with the way the surgery went today”), confidence (“I am sure that the joint function has been normalized as well as possible”), perceived workload using a reduced version of the National Aeronautics and Space Administration Task Load Index (NASA TLX) [18, 19] (“Working with the robotic arm was not physically stressful for me”), team interactions (“The team communication was very good today”), and team familiarity (“The team interaction with the robotic arm worked very well today”).”

Categorical data were compared using the Chi² test. Normal distribution of all data sets was assessed with the Kolmogorov-Smirnov test. To assess team member-specific differences within surgical steps, the Kruskal-Wallis Test with Dunn’s multiple comparison test was used. Statistical significance was set at a p-value < 0.05 for all analyses. All statistical analysis was performed using GraphPad Prism (Version 10.0, GraphPad Software, Inc., La Jolla, US-CA).

## Results

Preoperative Stress Assessment showed that there was no significant difference between different team members regarding the STAI-6 score (Table 1, *p* = 0.202). The technician showed the highest number in severe anxiety (9 cases 15%), while scrub techs showed the lowest number in severe anxiety (5 cases, 8%), followed by the surgeons (6 cases 10%). However, all team members showed no anxiety in more than 80% of the cases imediatelybefore surgery.

When considering the overall team stress level, only in 2 cases a severe stress level was measured (3%), while in 57 cases the team showed no anxiety (95%). No statistically significant difference could be found within different team configurations divided by the lead surgeon (*p* = 0.229).

**Table 1:** Preoperative assessed stress level using the STAI-6 score reported for the 60 surgeries for the 3 different roles: surgeons, scrub techs, and technicians. Median team stress level per surgery was calculated and reported for all surgeries, as well as for the three different team configurations depending on the lead surgeon (team surgeon 1, team surgeon 2, and team surgeon 3)

STAI-6 Stress level	no anxiety	moderate anxiety	severe anxiety
Surgeons	52 (87%)	2 (3%)	6 (10%)
Scrub techs	51 (85%	4 (7%)	5 (8%)
Technician	50 (83%)	1 (2%)	9 (15%
Median between all team members	57 (95%)	1 (2%)	2 (3%)
Median team surgeon 1	18 (95%)	0 (0%)	1 (5%)
Median team surgeon 2	14 (93%)	0 (0%)	1 (7%)
Median team surgeon 3	25 (95%)	1 (4%)	0 (0%)

Overall cut-to-suture time averaged 1 h 30 min (range 1:00–2:37), with no significant differences between the three surgical teams (*p* = 0.247). Median times were 1:39 for surgeon 1 (range 1:04–2:37), 1:38 for surgeon 2 (1:03–1:54), and 1:23 for surgeon 3 (1:00–2:24).

No surgeon specific difference in the *HR*_norm_ measurements of scrub techs, technicians, and circulators could be detected (scrub techs *p* = 0.091, technicians *p* = 0.224, circulators *p* = 0.373).

*HR*_norm_ assessment during surgeries showed statistically significant differences between different surgical steps for all four team members (*p* < 0.001) equipped with HR sensors (Surgeons, scrub techs, technicians, and circulators).

**Fig. 1:**
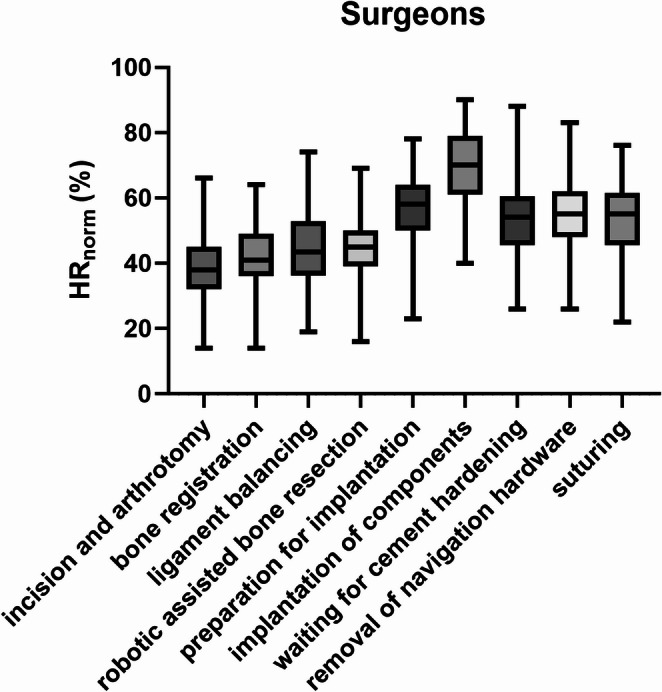
*HR*_norm_ for the three surgeons divided by surgical steps. Bars indicate the minimum and maximum values

Surgeons showed the highest *HR*_norm_ rate during the implantation of components in comparison to all other steps (*p* < 0.001) except for the preparation of implantation (*p* = 0.092) (Fig. 1). During the preparation for implantation, surgeons showed a statistically significantly higher *HR*_norm_ in comparison to all other steps (*p* < 0.001), except for the steps after the implantation (waiting for cement hardening, removal of navigation hardware, and suturing, *p* > 0.999) (Fig. 1). *HR*_norm_ of the surgeons was lowest during incision and arthrotomy differing significantly from preparation for implantation, implantation of components, waiting for cement hardening, removal of navigation hardware, and suturing (*p* < 0.001), but not from robotic phases (bone registration, ligament balancing, robotic bone resection, *p* > 0.641). (Fig. 1). The robotic steps did not differ among themselves (*p* > 0.999). (Fig. 1). *HR*_norm_ of the surgeons during ligament balancing was significantly lower than during the post-implantation steps (cement hardening, hardware removal, suturing) (*p* < 0.013), while these four post-implantation and preparation steps did not differ from each other (*p* > 0.999)(Fig. 1).

**Fig. 2:**
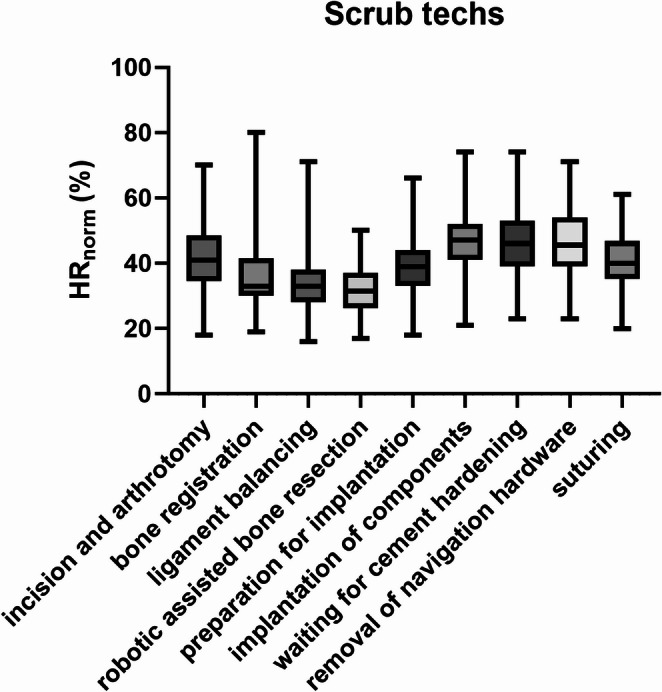
*HR*_norm_ for the scrub techs divided by surgical steps. Bars indicate the minimum and maximum values

For scrub techs *HR*_norm_ was lowest during robotic assisted bone resection, statistically significant lower than all other steps (*p* ≤ 0.020) but not different from the robotic specific steps, bone registration, and ligament balancing (*p* > 0.999) (Fig. 2). Ligament balancing showed a lower *HR*_norm_ for scrub techs than incision and arthrotomy, preparation for implantation, implantation of components, waiting for cement hardening (, removal of navigation hardware, and suturing (*p* ≤ 0.005) (Fig. 2). Bone registration yielded lower*HR*_norm_ of scrub techs thanimplantation of components, waiting for cement hardening, and removal of navigation hardware (*p* < 0.001) (Fig. 2). *HR*_norm_ of scrub techs increased significantly from preparation of implantation to implantation of components (*p* = 0.038), and then remained stable through waiting for cement hardening, removal of navigation hardware, suturing, (*p* > 0.092) (Fig. 2).

**Fig. 3:**
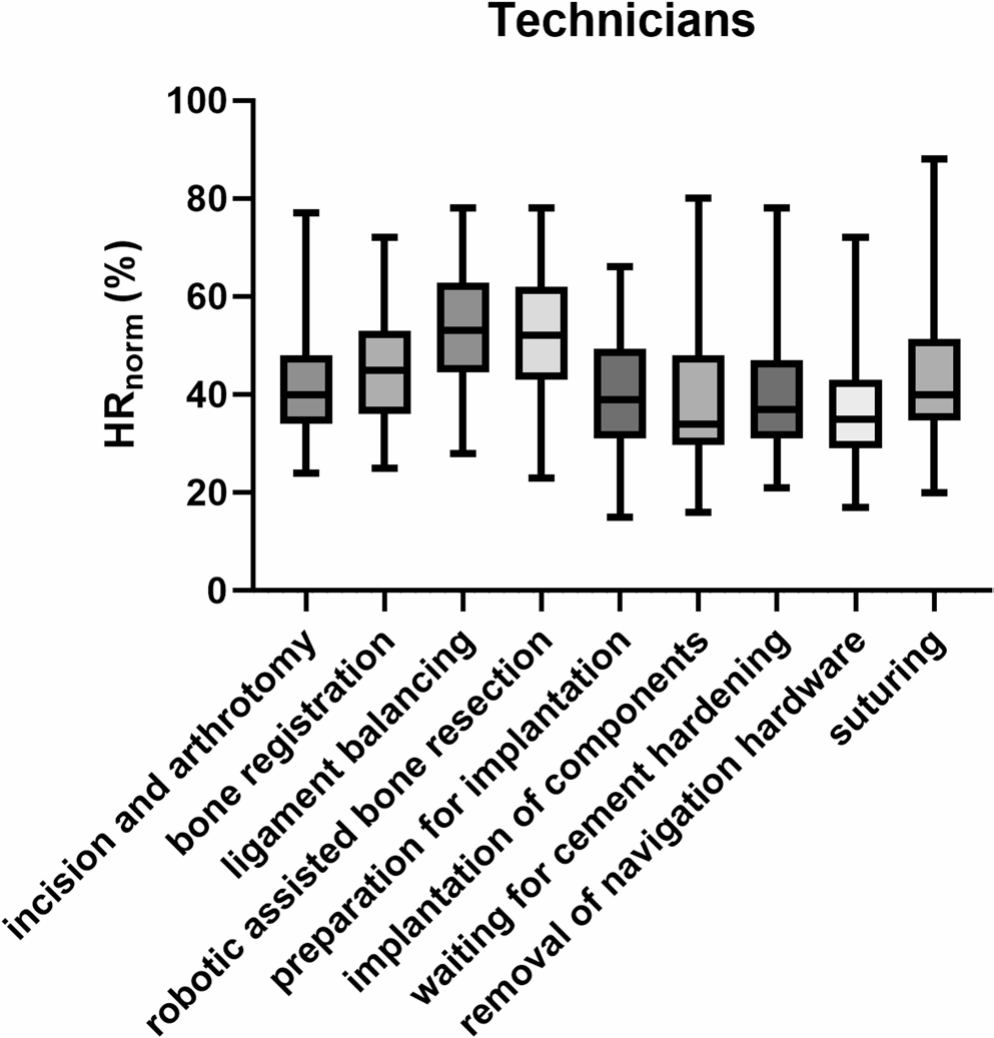
*HR*_norm_ for the technicians divided by surgical steps. Bars indicate the minimum and maximum values

Technician showed their highest *HR*_norm_ during ligament balancing significantly exceeding all non-robotic steps (*p* ≤ 0.001) and not differing from robotic assisted bone resection (also high) (*p* < 0.999), and bone registration (*p* = 0.106) (Fig. 3). Robotic assisted bone resection similarly showed higher *HR*_norm_ of technicians than all non-robotic steps (*p* ≤ 0.011) and did not differ from robotic assisted bone resection and bone registration (*p* = 0.714) (Fig. 3). During bone registration, *HR*_norm_ of technicians was higher than in implantation of components (*p* = 0.004) and removal of navigation hardware (*p* = 0.003), while all othercomparisons showed no significant differences (*p* > 0.237) (Fig. 3).

**Fig. 4:**
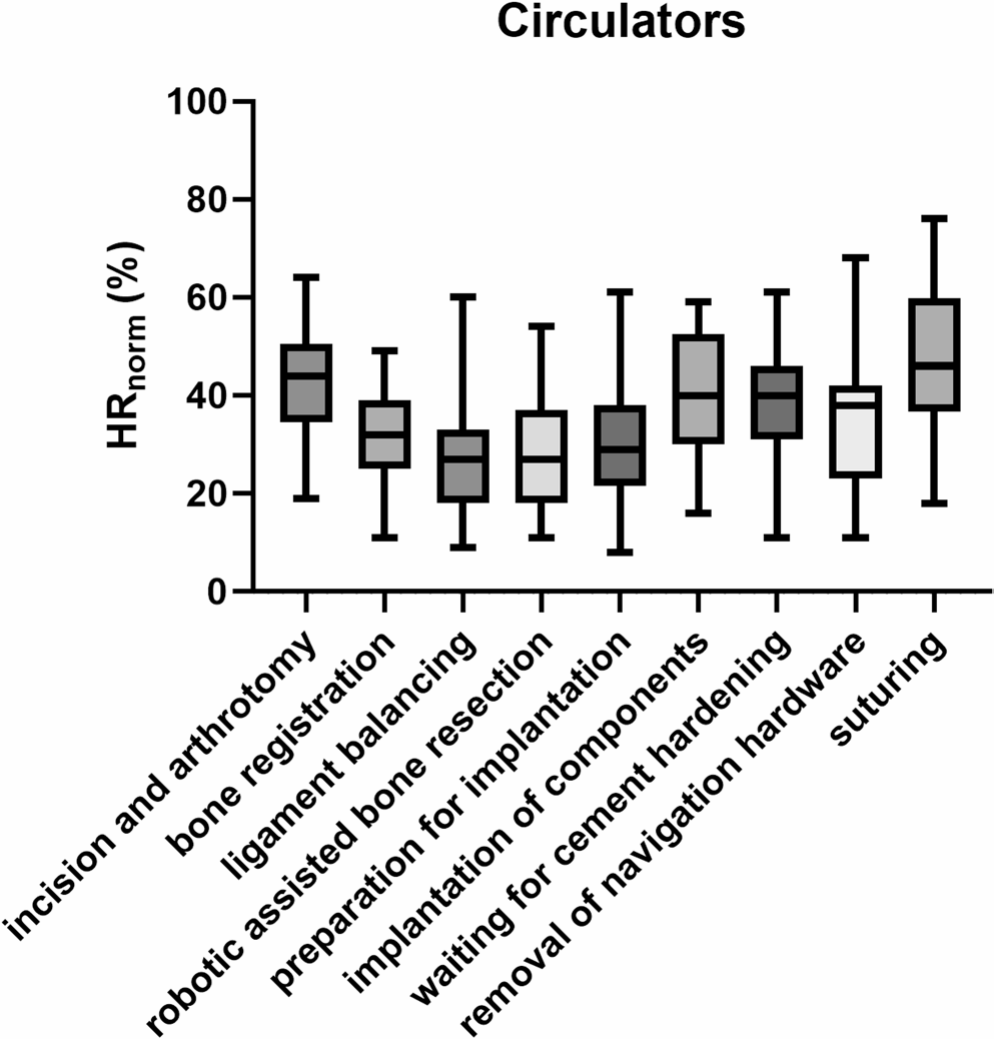
*HR*_norm_ for the technicians divided by surgical steps. Bars indicate the minimum and maximum values

Circulators showed the highest *HR*_norm_ during suturing, significantly exceeding bone registration, ligament balancing, robotic assisted bone resection, and preparation for implantation (*p* ≤ 0.002) (Fig. 4). Their lowest *HR*_norm_ occured during ligament balancing and robotic assisted bone resection (Fig. 4). Ligament balancing showed lower *HR*_norm_ of circulatorsthan incision and arthrotomy (*p* = 0.001) and implantation of components (*p* = 0.011) (Fig. 4). During the step incision and arthrotomy, a *HR*_norm_ was recorded comparable to the last phase of surgery with no statistically significant difference to implantation of components, waiting for cement hardening, removal of navigation hardware, and suturing (*p* > 0.875) (Fig. 4). No difference of *HR*_norm_ of the circulator was found between bone registration and incision and arthrotomy (*p* = 0.069) (Fig. 4). During incision and arthrotomy a higher *HR*_norm_ of circulators was assessed than in robotic assisted bone resection (*p* = 0.001) and preparation for implantation (*p* = 0.008) (Fig. 4). A statistically significant lower *HR*_norm_ was found for robotic assisted bone resection in comparison to the implantation of components (0.027) (Fig. 4). All other pairwise comparisons were not significant.

**Table 2:** Normalized median *HR*_norm_ values in % with range in brackets for the team members divided by surgical steps

Surgical step	Surgeons	Scrub techs	Technicans	Circulators	*P*-Value
Incision and arthrotomy	38 (14–66)	41 (18–70)	40 (24–77)	44 (19–64)	0,305
Bone registration	41 (14–64)	33 (19–80)	45 (25–72)	32 (11–49)	< 0,001
Ligament balancing	43 (19–74)	33 (16–71)	53 (28–78)	27 (9–60)	< 0,001
Robotic assisted bone resection	45 (16–69)	31 (17–50)	52 (23–78)	27 (11–54)	< 0,001
Preparation for implantation	58 (23–78)	39 (18–66)	39 (15–66)	29 (8–61)	< 0,001
Implantation of components	70 (40–90)	47 (21–74)	34 (21–74)	40 (16–59)	< 0,001
Waiting for cement hardening	54 (26–88)	46 (23–74)	37 (21–78)	40 (11–61)	< 0,001
Removal of navigation hardware	55 (26–83)	45 (23–71)	35 (17–72)	38 (11–68)	< 0,001
Suturing	55 (22–76)	40 (20–61)	40 (20–88)	46 (18–76)	< 0,001

Across team members, a statistically significant difference was determined in all steps except for the incision and arthrotomy (Table 2). Scrub techs and circulators showed no difference in *HR*_norm_ (*p* > 0.054) except for the removal of navigation hardware, where circulators had a statistically significant higher *HR*_norm_ (*p* = 0.005).

In bone registration surgeons and technicians had similarly high *HR*_norm_ (*p* > 0.999), and both exceeded scrub techs and circulators (*p* ≤ 0.004).

During ligament balancing, technicians had the highest *HR*_norm_ followed by surgeons (*p* = 0.017), both significantly higher than, scrub techs (*p* < 0.001), and circulators (*p* < 0.001).

During robotic bone resection, surgeons and technicians again showed comparable, elevated *HR*_norm_ (*p* = 0.151), exceeding scrub techs and circulators (*p* < 0.001). Surgeons showed a statistically significant higher *HR*_norm_ during preparation of implantation (*p* < 0.001), during implantation of components (*p* < 0.001), during waiting for cement hardening (*p* < 0.039), and removal of navigation hardware (*p* < 0.039) than all other team members. During suturing, surgeons *HR*_norm_ exceeded scrub techs and technicians (*p* < 0.001) but not circulators (*p* = 0.280). Technicians showed higher *HR*_norm_ than circulators during preparation (*p* = 0.031), while there was no statistically significant difference between technicians and scrub techs (*p* > 0.999).

Scrub techs showed a statistically significant higher *HR*_norm_ than technicians during implantation of components, waiting for cement hardening, removal of navigation hardware (*p* ≤ 0.024), but not suturing (*p* > 0.999). In these four surgical steps (implantation of components, waiting for cement hardening, removal of navigation hardware, suturing technicians and circulators did not differ (*p* > 0.705).

Considering the questionnaire about individual satisfaction, confidence, workload, team interaction, and team familiarity, no statistically difference could be found between the three teams (*p* = 0.381) (Table 3). However, all team members of surgeon 2 showed a very high satisfaction in all cases and confidence, team familiarity, and team interaction were rated with the highest score in more than 90% of the cases (Table 3). Workload was reported to be low in 68% of the cases by the team members of surgeon 1 (Table 3). A statistically significant difference could be found between the surgeons, scrub techs and technicians regarding the questions of the postoperative questionnaire (*p* < 0.001). Technicians showed in 69% of cases the highest satisfaction, while surgeons and scrub techs were highly satisfied in around 60% of cases (Table 3). Scrub techs felt highly confident in 85% of cases, while technicians in 80% and surgeons in 70% of cases (Table 3). The workload was acceptable for technicians in 83% of cases and for surgeons in 70% of cases, while the workload was acceptable for scrub techs only in 20% of cases (Table 3).

Surgeons were very satisfied with the team interaction in 92% of cases, while scrub techs were satisfied in 83% and technicians in 75% of cases. Regarding team familiarity, surgeons gave the highest score in 85% of the cases, while scrub techs and technicians gave in around 73 to 75% the cases (Table 3).

**Table 3:** Number of times in % the highest score was achieved for each question in the postoperative questionnaire

	Overall team	Team Surgeon 1	Team Surgeon 2	Team Surgeon 3	Surgeons	Scrub techs	Technicians
**Satisfaction**	67	58	73	69	63	61	69
**Confidence**	87	74	100	88	70	85	80
**Workload**	57	68	53	50	70	20	83
**Team interaction**	92	79	100	96	92	83	75
**Team familiarity**	83	74	93	85	85	73	75

## Discussion

This study examined the influence of modern robot technology on the surgical staff. It was found that the average HR of all staff members underwent significant changes during several steps of the robot-assisted TKA.

Preoperatively, Stress Assessment was performed using the STAI-6 questionnaire. Overall, all team members showed no anxiety in 80% of the cases no anxiety before the surgery. Scrub techs, as well as surgeons, who were newly introduced to the system, showed a severe anxiety in 8% (scrub techs) and 10% (surgeons) of the cases, while technicians showed a severe anxiety in 15% of cases. The introduction of new technology in the surgical workflow might therefore also trigger a higher preoperative stress level. For the technician, besides having less OR experience, he was also introduced newly to the team, which might explain the higher stress level preoperatively, although no statistically significant difference could be found between different team members. Preoperative measured stress level was also independent of the lead surgeon, as no statistically significant difference between different teams could be found. The number of cases with preoperative severe stress levels for the surgeons is well in line with the initial learning phase of the surgical technique, which can take up to 15 cases [13, 21, 22]. However, higher stress levels were also observed after the initial learning curve as a particularly difficult case might also raise the preoperative stress level for the whole team. Preoperative stress levels of scrub techs were measured throughout the whole group of 10 different scrub techs. Out of this group, not all study participants reported severe stress levels preoperatively, which might indicate that most of them felt well trained and prepared for the surgery.

Cut to suture time was 1 h and 30 min (range 1 h to 2 h and 37 min), which is well in line with times reported in other studies [15, 21]. No statistically significant difference was found between the three different teams in terms of cut to suture time (*p* = 0.247).

Intraoperative stress levels were assessed using normalized *HR*_norm_. No surgeon specific difference in the *HR*_norm_ measurements of scrub techs, technicians, and circulators could be detected (scrub techs *p* = 0.091, technicians *p* = 0.224, circulators *p* = 0.373), which could also be observed in the preoperative questionnaires. The stress level of the single team members was not affected by an individual surgeon.

The most demanding surgical step with the highest *HR*_norm_ for surgeons was measured during the implantation of components and during the preparation for implantation. As this step is performed within every surgery, and all three surgeons were very experienced, the increased *HR*_norm_ might be explained mostly by physical stress. The *HR*_norm_ lowered after the implantation steps during the waiting for cement hardening, removal of navigation hardware, and suturing. We expected a higher decline of the *HR*_norm_ during the waiting for the cement hardening process, however the data did not show such behavior. The stress level for the last phase of the surgery (waiting for cement hardening, removal of navigation hardware, and suturing) was statistically significant higher than during the initial phase (incision and arthrotomy). As the activity level might be similar between the initial phase and final phase of the surgery, the higher *HR*_norm_ might be explained by a longer cool-down phase or an increased psychological stress level at the end of the surgery. It might also be that the *HR*_norm_ is not sensitive enough to detect small stress level variations.

Surprisingly, surgeons showed a similar *HR*_norm_ within the newly introduced robotic specific steps, which were higher than during the initial phase (incision of arthrotomy) but lower than all other steps performed after the robotic assisted cutting procedure. This might indicate that the robotic arm itself did not create any additional physical or physiological stress for the surgeon itself as it might be considered simply a different tool used to perform the surgery.

The impact of the robotic arm is also visible in the *HR*_norm_ curves of scrub techs and circulators. Both show a higher *HR*_norm_ during the not robotic arm assisted steps, where more physical activity of the team members was necessary. Scrub techs as well as circulators show a high activity during the implantation of components, well in line with the surgeons. For the circulators, *HR*_norm_ levels change as they have some resting phases and high activity phases, while the scrub techs maintain a high activity level throughout the surgery, except for the robotic assisted steps. This might be an indication that the stress level for scrub techs and circulators is getting reduced with the usage of a robotic arm.

In contrast to that, the stress level of the technician rises and reaches the highest HR level during ligament balancing and robotic assisted bone resection. The activity level of the technician itself is moderate as he is responsible only for the robot and usually not take over any other work activities in the OR. The presence of a dedicated robotic technician—tasked with system setup, calibration, and real-time intraoperative support—substantially alleviates the cognitive and technical workload of surgeons and operating room staff, facilitating smoother case transitions and improving intraoperative workflow [6]. Fontalis et al. highlights the technician’s pivotal role in troubleshooting and optimizing procedural efficiency [23]. The highest activity level of the technician was observed when maneuvering the robotic arm into the cutting position and when removing it. During ligament balancing, the increased *HR*_norm_ of the technician might reflect the physiological stress level due to the interaction with the software and the critical step of repositioning the implant according to the assessed ligament tension. This step might be considered the most critical step for the technician, as a misplaced implant can provoke significant patient harm, which might explain the increased *HR*_norm_ for this specific step.

Circulators showed the highest *HR*_norm_ during the surgical step of suturing, as they usually start by removing tools and starting the cleaning up process. They also might push in a mobile C-arm for postoperative X-rays. The *HR*_norm_ during incision is comparable to the initial phase of the surgery, where the circulator is still bringing in components and helping set up the surgery. Activity level remains high also during preparation for implantation, final implantation of components, waiting for cement hardening, and removal of navigation components.

When comparing the *HR*_norm_ of the team members between each other during the incision and arthrotomy no difference could be observed. During the progress of the surgery, circulators and scrub techs showed no significant difference between each other except for the final phase of the surgery (removal of navigation hardware), where circulators start to clean up and remove items. In the robotic specific step bone registration no statistically significant difference was found between surgeons and technicians, while surgeons and technicians had a statistically higher *HR*_norm_ in comparison to scrub techs and circulators. The introduction of the robotic arm mainly affects the surgeon itself as well as the newly introduced profession of the technician. During ligament, balancing the highest *HR*_norm_ was recorded for the technicians in comparison to surgeons, which might indicate a higher stress level due to the importance of the step, as the technician adapts the implant position on behalf of the surgeons indications. An algorithmic based positioning of the implant might help in reducing the stress level within this step as well as additionally safety features to reduce potential patient harm as much as possible. It has to be pointed out that the technician was the least experienced team member in the OR, which had to interact with a more experienced group, which performed TKA surgeries on a daily base.

Surgeons showed a statistically significant higher *HR*_norm_ during preparation of implantation, during implantation of components, while waiting for cement hardening and removal of navigation hardware than all other team members. It can be deduced that the most critical step for the surgeon is the insertion of the implant, which is probably also the most physically demanding step. Also, the usage of the robotic arm forces the surgeon to follow a software based workflow with some steps, which are physically less demanding (e.g. bone registration). As soon as the cuts are being performed and the robotic arm is being removed, the surgeon can again follow his preferred workflow and influence the speed of the operation, which might be reflected also on a higher *HR*_norm_ as well as a higher activity level. Indeed, also scrub techs show a higher *HR*_norm_ activity in the non robotic assisted steps in the second phase of the surgery.

Postoperative questionnaires showed no difference between teams led by the three different surgeons. The higher satisfaction and confidence level as well as high team familiarity and team interaction can be explained as surgeon 1 and 3 were doing on one hand more cases than surgeon 2 and on the other hand performed also the initial cases, where the team was getting trained. Workload was reported acceptable in only around half of the cases. A statistically significant difference could be found between the surgeons, scrub techs and technicians regarding the questions of the postoperative questionnaire. All team members were highly satisfied in around 60% of the cases. Scrub techs fell highly confident in 85% of the cases, while technicians in 80% and surgeons in 70% of the cases. The workload was acceptable for technicians in 83% of cases and for surgeons in 70% of cases while the workload was acceptable for scrub techs only in 20% of cases. The workload of the technicians was somehow surprising as their activity level should be lower than standard total knee surgeries as some workload is transferred to the technician. On the other hand, during the preparation procedure the scrub techs have to perform additional steps (calibration of the robotic arm, draping of the robotic arm) and prepare additional instruments (robotic specific instruments), which might explain the increased reported workload. It has to be pointed out that scrub techs did prepare all the standard alignment tools due to requirements of the internal quality assurance system, although this was not required in a robotic assisted surgery. Martinello et al. reported that nursing staff face significant challenges in adapting to robotic surgery, primarily related to the unfamiliar setup process, expanded intraoperative responsibilities, and prolonged operative times [24]. To facilitate this transition, most manufacturers offer structured training programs designed to minimize variability in team performance during the early stages of implementation [25]. Moreover, the integration of a dedicated robotic technician — responsible for system setup, calibration, and real-time intraoperative support — can substantially reduce the cognitive and technical demands placed on surgeons and operating room staff [6]. This support enables smoother case transitions and improves intraoperative workflow. Fontalis et al. particularly emphasize the technician’s essential role in troubleshooting and optimizing procedural efficiency [23].

Surgeons were very satisfied with the team interaction in 92% of cases, while scrub techs were satisfied in 83% and technicians in 75% of cases. This might reflect the point that the technician was the newest member, which was not fully integrated in the clinic. Team familiarity reached the highest scores in more than 70% of the cases by all team members.

No comparison group to a conventional TKA was assessed, which limits the results to robotic assisted TKA only and to the specific robotic-arm used in the study. However the surgical steps incision and arthrotomy, preparation for implantation, implantation of components, waiting for cement hardening as well as suturing would be performed in the same way also in conventional TKA. Other robotic surgical systems used in TKA do not require a technician to guide the robotic arm, which might result in different *HR*_norm_ patterns during surgery.

The study reports data from the initial learning phase during the introducing of a robotic system. Therefore, some stress related patterns might be different in a routine surgery. However, the dataset consists of 60 cases and therefore the majority of cases should be outside of the initial learning curve reported from literature [13, 21, 22]. Questionnaires are subjective measurements that might be biased by the study participants. The majority of the cases were performed by one single technician. Questionnaires of the surgical team might be less biased as they reflect the opinions from three different surgeons and 10 different scrub techs. Circulators were excluded from the questionnaires, as many times there were personal changes within the surgery. Personal rotation occurred also in some cases for the scrub techs, therefore not all the data was recorded in the HR measurements. In two measurements the sensor did not get al.l data points (probably it was fixed to loose on the upper arm). The change in HR rate measurements is not very sensitive; therefore, the works steps were divided in easy distinguishable tasks.

Another limitation of the study is that HR measurements are not distinguishing between physical and psychological stress. Arora et al. demonstrated that reliance solely on an objective marker such as heart rate limits the validity and reliability of findings, since it precludes differentiation between physical exertion and psychological stress [9]. A multimodal assessment approach, integrating both physiological and subjective indicators, has therefore been proposed as a more robust method for evaluating intraoperative stress [9]. To overcome this limitation, we used subjective questionnaires to assess the STAI-6 score.

## Conclusion

This study provides novel insights into the impact of robotic-assisted technology on intraoperative stress and workload among surgical staff during total knee arthroplasty. Heart rate analysis revealed that stress levels varied significantly across surgical steps, with the highest demand for surgeons during component implantation, while scrub technicians and circulators exhibited increased activity during non-robotic phases. In contrast, technicians experienced the greatest stress during robot-specific steps, particularly ligament balancing, reflecting the criticality of their role and their relative inexperience.

Preoperative assessments demonstrated generally low anxiety levels, although newly introduced staff—particularly technicians—reported higher stress, likely attributable to limited experience and integration into the team. While postoperative questionnaires indicated high satisfaction, confidence, and team familiarity, scrub technicians consistently reported a disproportionate workload, reflecting the additional tasks required for robotic preparation.

Importantly, the robotic arm itself did not impose additional physiological stress on surgeons and appeared to reduce workload for scrub technicians and circulators, while shifting critical responsibility to the technician. These findings highlight that robotic assistance redistributes intraoperative stress rather than eliminating it.

## Data Availability

Due to privacy restrictions the data will be available on request of the corresponding author.

## Declarations

### Permission to reproduce material from other sources

Not required.

### Clinical trial registration

Not required.
